# Mechanisms of metabolic transition in hypertrophic cardiomyopathy

**DOI:** 10.3389/fphys.2025.1700313

**Published:** 2025-11-12

**Authors:** Conghao Tan, Zhexuan Guo, Junjie Zhou, Wei Yuan

**Affiliations:** 1 Department of Cardiology, Affiliated Hospital of Jiangsu University, Zhenjiang, China; 2 Institute of Cardiovascular Diseases, Jiangsu University, Zhenjiang, China

**Keywords:** hypertrophic cardiomyopathy, metabolic transition, mitochondrial dysfunction, glucose metabolism, lipid metabolism

## Abstract

Hypertrophic cardiomyopathy (HCM) is a prevalent hereditary cardiovascular disease that affects individuals worldwide. While current treatments have improved the prognosis for many patients, HCM continues to impose a significant burden on global healthcare systems. Understanding its underlying mechanisms, particularly the role of metabolic transition, is crucial for enhancing diagnosis and treatment strategies. One of the most promising areas of research in HCM is the study of metabolic transition. This process, which involves significant changes in energy production and consumption within cardiac cells, has become increasingly recognized as a key factor in the disease’s progression. In HCM, glucose metabolism is markedly altered. The heart increasingly relies on glycolysis for energy production, while the aerobic oxidation of glucose is impaired. These changes are accompanied by alterations in the activity of glucose transporter proteins and key enzymes involved in glycolysis. Such abnormalities are closely associated with myocardial ischemia, fibrosis, and an increased risk of acidosis in cardiomyocytes, which in turn affects calcium cycling and cardiac diastolic function. Lipid metabolism is significantly altered in HCM. There is a defect in fatty acid β-oxidation, leading to the accumulation of ceramides and sphingomyelins in cardiomyocytes. Additionally, changes in ketone body metabolism occur as an adaptive response to energy deficiency, which may further affect cardiac function. Amino acid metabolism is also altered in HCM. Elevated levels of branched-chain amino acids have been observed, and these metabolites are strongly associated with cardiac remodeling and the development of insulin resistance. These changes further contribute to the maladaptive processes in HCM. A comprehensive understanding of the metabolic transition process in HCM is essential for unraveling the disease’s pathogenesis. Such insights could pave the way for novel therapeutic strategies, ultimately improving patient outcomes and quality of life.

## 1 What is hypertrophic cardiomyopathy?

Hypertrophic cardiomyopathy (HCM) is a heterogeneous group of cardiovascular diseases primarily driven by genetic mutations (Braunwald et al., 1964; Braunwald, 2024). This genetic diversity complicates both diagnosis and treatment, with mutations in key genes, such as β-myosin heavy chain and myosin-binding protein C, playing a central role in the disease’s development (Ingles et al., 2015; Mohamed et al., 2017; Hsieh et al., 2022). HCM is characterized by non-load-dependent myocardial hypertrophy, defined by a left ventricular wall thickness of ≥15 mm in any myocardial segment (Batzner and Seggewiß, 2020; Ommen and Semsarian, 2021). This hypertrophy occurs without any evident changes in blood pressure or heart valve function, distinguishing it from other conditions where hypertrophy is caused by such external factors. The clinical manifestations of HCM vary widely. In mild cases, individuals may remain asymptomatic or experience only a slight reduction in exercise tolerance. However, in more severe cases, the disease can lead to significant cardiac dysfunction, including heart failure, left ventricular outflow tract obstruction (LVOT), and an increased risk of sudden cardiac death. Other complications such as syncope, malignant arrhythmias, and myocardial ischemia are also common in advanced stages (Finocchiaro et al., 2020; D’Onofrio et al., 2023; Ommen et al., 2024). Recent studies have revealed that, in addition to genetic mutations, abnormal cell signaling pathways and metabolic dysfunction play a critical role in HCM’s progression. These factors contribute to the disease’s pathophysiology by altering myocardial energy metabolism and cellular processes, exacerbating cardiac remodeling, and leading to functional impairment (Beghini et al., 2024). Although current clinical interventions have significantly improved the overall prognosis for many HCM patients, the disease still represents a substantial burden on healthcare systems. The need for frequent hospitalizations, specialized care, and the high costs of managing complications such as heart failure and arrhythmias contribute to the ongoing socioeconomic challenges associated with HCM (Butzner et al., 2021).

### 1.1 Epidemiologic characteristics of HCM

Global burden of disease studies indicate that cardiovascular diseases, including HCM, impose a particularly high burden in low- and middle-income countries, despite growing awareness of their health impact worldwide (Lavie, 2023). HCM is one of the most common hereditary cardiovascular diseases, with an estimated prevalence of approximately 0.2% in the general population, contributing to a substantial disease burden globally (Maron et al., 1995; Massera et al., 2023). The growing prevalence of HCM has significantly increased the disease burden on healthcare systems. In the United States, for instance, the number of clinically diagnosed HCM patients is expected to rise by 1.5 times, reaching an estimated 262,591 cases based on data from the HealthCore Integrated Research Database (HIRD) (2013–2019) (Butzner et al., 2021). Of these, approximately 75% of HCM patients have obstructive HCM (oHCM), which is characterized by LVOT obstruction, leading to significant clinical implications, including increased risk of heart failure and arrhythmias (Lu et al., 2018). A large analysis of U.S. claims data (n = 11,401) revealed that the all-cause annual medical costs for symptomatic oHCM patients averaged $43,586 per case, nearly six times higher than the $6,768 per case for matched healthy controls. Additionally, the annual hospitalization rate for symptomatic oHCM patients (38%) is 5.5 times higher than for non-cardiomyopathic controls (6.9%), contributing to an extra $35,000 in annual medical expenditures per patient (Jain et al., 2021). A longitudinal study involving 4,591 patients from 8 HCM centers revealed that, during a median follow-up of 2.9 years, 22% of patients developed heart failure, 20% developed atrial fibrillation, and 6% reached the composite endpoint of sudden cardiac death (Semsarian et al., 2015). A high-screening cohort study also found that the annual mortality rate for patients with HCM ranges from 3% to 6%, further underscoring the significant clinical impact of the disease (Younger et al., 2020). Although the clinical phenotype of HCM shows minimal variation across different racial groups (Butters et al., 2021), gender differences are notable. Female patients often present with a later age of onset, more severe disease, and worse survival outcomes compared to their male counterparts (Geske et al., 2017).

### 1.2 Pathogenesis of hypertrophic cardiomyopathy

Numerous studies have established that the pathogenesis of HCM is strongly influenced by genetic factors, typically following an autosomal dominant inheritance pattern, often involving complex genetic interactions (Teekakirikul et al., 2019). Clinical data reveal that mutations in known causative genes are detectable in approximately 60% of HCM patients, providing crucial insights into the genetic underpinnings of the disease (Hensley et al., 2015; Marian and Braunwald, 2017; Maron et al., 2019). The majority of these causative mutations are found in genes encoding cardiac myosin, including ACTC1, MYBPC3, MYH7, MYL2, MYL3, TNNI3, and TNNT2, which are central to the molecular pathogenesis of HCM. Over 1,000 distinct genetic variants have been identified within these genes, highlighting the genetic complexity and variability of HCM (Niimura et al., 1998; Alfares et al., 2015). Mutations in these genes lead to a series of pathophysiological changes, beginning with abnormal calcium handling within cardiac cells, followed by increased myofilament stress and dysfunction in energy metabolism and mitochondrial function (Marian and Braunwald, 2017; Menezes Junior et al., 2024). These disturbances ultimately contribute to the development and progression of HCM. In addition to genetic factors, mutations in mitochondrial DNA, metabolic abnormalities, and other environmental or acquired factors also contribute to the progression of HCM (Elliott, 2018; Hayashi, 2020). Mitochondrial dysfunction is a central and pervasive feature of HCM pathophysiology, playing a crucial role in disease progression, regardless of the underlying genetic or environmental cause (Viola and Hool, 2019). These molecular disturbances disrupt intracellular calcium homeostasis, interfere with cellular metabolism, and lead to the production of reactive ROS such as superoxide anions, hydroxyl radicals, and peroxyl radicals (Bhatti et al., 2017; Wasmus and Dudek, 2020; Weissman and Maack, 2021). This generation of ROS plays a critical role in the pathogenesis of HCM, contributing to further cellular damage and disease progression.

### 1.3 Detection methods of HCM

Electrocardiography (ECG) is a fundamental and accessible tool commonly used in the initial evaluation of patients suspected of having HCM(13). Owing to its simplicity, cost-effectiveness, and wide availability, ECG remains a standard component of cardiovascular assessment and can offer important diagnostic and prognostic clues in HCM (8). There is an emerging interest in artificial intelligence-enhanced 12-lead electrocardiogram (AI-ECG) in detection of HCM (14,15). However, ECG findings are often nonspecific, and approximately 4%–6% of adult patients may even present with normal ECGs. This variability limits ECG’s reliability as a standalone diagnostic method (16,17). Transthoracic echocardiography remains the cornerstone of HCM diagnosis, offering direct visualization of asymmetric septal hypertrophy and LVOT obstruction (18). It also plays a critical role in guiding therapeutic planning, including decisions regarding septal reduction therapy or surgical intervention (9). Cardiac magnetic resonance imaging (CMR) is particularly valuable when echocardiographic findings are inconclusive or when more precise myocardial characterization is needed. Its superior spatial resolution and tissue contrast allow for the detailed assessment of myocardial fibrosis, ventricular morphology, and hemodynamics. Various CMR techniques have been widely applied for phenotypic classification, risk stratification, and therapeutic decision-making in patients with HCM (19,20). Genetic testing has emerged as an important adjunct in the comprehensive evaluation of HCM, especially in familial or ambiguous presentations. Identification of mutations in sarcomeric protein genes provides definitive molecular evidence to support the diagnosis. Furthermore, genetic testing plays a crucial role in differentiating HCM from secondary forms of cardiac hypertrophy, such as those due to chronic hypertension, valvular disease, or ischemic cardiomyopathy (21,22).

## 2 Relationship between HCM and metabolic transition

### 2.1 What is metabolic transition?

In contrast to the adult heart, the fetal heart operates in a relatively hypoxic environment, with lower levels of fatty acids in the circulatory system. Additionally, the energy demand of the fetal heart is lower due to its reduced workload during this developmental stage. During this period, fetal cardiomyocytes primarily rely on anaerobic glycolysis and lactate oxidation for energy production, as the low oxygen environment limits the ability to utilize oxidative phosphorylation (Lopaschuk and Jaswal, 2010). As the perinatal period begins, the metabolic pattern of cardiomyocytes shifts dramatically from carbohydrate-based energy production to fatty acid oxidation. This shift is accompanied by an increase in mitochondrial oxidative capacity and a corresponding rise in fatty acid oxidative flux. As HCM progresses, the ATP generation mechanism of cardiomyocytes undergoes adaptive remodeling. This is characterized by a decrease in fatty acid oxidation, an increase in glycolysis, and the activation of alternative metabolic pathways. There is a marked increase in the utilization of alternative energy sources, such as lactate, branched-chain amino acids (BCAAs), and ketone bodies (Neubauer, 2007; Vega et al., 2015). This shift in metabolic patterns back toward those seen in fetal life is known as metabolic reprogramming. It reflects a reversion to less efficient energy production pathways, which contribute to disease progression. Metabolic reprogramming is driven by the dynamic regulation of core metabolic pathways, including glycolysis, fatty acid oxidation, amino acid metabolism, and mitochondrial function. This process involves a reconfiguration of the metabolic network at multiple levels to adapt to the disease environment.

### 2.2 Effects of cardiac metabolic reprogramming on the body

Cardiac metabolic reprogramming is crucial for maintaining cardiac homeostasis and plays an important role in regulating systemic energy metabolism and body weight balance. A complex network of metabolic signaling interactions exists between the heart and peripheral organs. Evidence suggests that the heart regulates metabolic processes in other organs, such as adipose tissue and the liver, by secreting factors like natriuretic peptides that influence cellular pathways (Nakamura and Sadoshima, 2014). For instance, cardiac natriuretic peptide (CNP) not only mediates urinary sodium excretion, diuresis, and vasodilation but also plays a role in metabolic regulation. CNP activates the lipolytic signaling pathway in adipocytes, promotes mitochondrial biosynthesis, and enhances energy expenditure, thereby contributing to overall metabolic balance (De Bold, 2011). When cardiac metabolic homeostasis is disrupted, cardiomyocytes compensate for impaired fatty acid oxidation by upregulating glycolysis. This adaptation helps to meet immediate energy demands but comes with long-term metabolic consequences. While glycolysis can rapidly generate ATP to meet short-term energy demands, it is far less efficient than fatty acid oxidation in terms of energy production. This compensatory shift in metabolism can lead to increased fat deposition in adipose tissue, activation of inflammatory responses, and elevated oxidative stress. These factors collectively contribute to adverse cardiovascular effects and disease progression (Fuster et al., 2016).

Metabolic diseases such as obesity and diabetes can disrupt the balance of adipokine secretion. This disruption results in an increase in pro-inflammatory adipokines and a decrease in anti-inflammatory adipokines, which triggers a chronic low-grade inflammatory state. Over time, this imbalance contributes to systemic metabolic dysfunction (Ouchi et al., 2011). Moreover, optimizing cardiometabolic regulatory networks plays a crucial role in modern health management strategies, as it enhances the body’s ability to maintain metabolic balance and prevent diseases related to metabolic dysfunction. Maintaining a healthy cardiac metabolic state is essential for disease prevention, weight management, and overall health enhancement. It forms the foundation for the normal functioning of the body’s physiological systems.

## 3 Metabolic transition in HCM

Under normal physiological conditions, cardiomyocyte energy supply is primarily derived from the metabolism of free fatty acids (FFAs), which account for approximately 60%–90% of the energy required. FFAs are oxidized in mitochondria to produce ATP, which is essential for maintaining normal heart function (Chen et al., 2024). The heart exhibits remarkable metabolic plasticity, allowing it to flexibly utilize multiple substrates depending on energy demand. However, in the pathological process of HCM, there is a significant shift in myocardial energy metabolism. The heart moves from an efficient energy production mode dominated by fatty acid oxidation to a less efficient mode that relies on glycolysis. Oxidative phosphorylation is the primary pathway for ATP production and occurs in the mitochondrial respiratory chain. In HCM patients, mitochondrial dysfunction is characterized by a significant decrease in the activity of respiratory chain complexes, leading to impaired electron transfer efficiency. This dysfunction also causes an abnormal increase in the generation of ROS, such as superoxide anions and hydrogen peroxide, during the electron transfer process. These mitochondrial dysfunctions contribute directly to a severe energy deficit in cardiomyocytes, impairing their ability to contract and function effectively (Christiansen et al., 2015; Viola and Hool, 2019). Dysfunctional oxidative phosphorylation not only limits ATP production but also exacerbates oxidative stress. This increased oxidative stress damages cardiomyocytes and surrounding tissue, further promoting the pathological progression of HCM (Table 1) (Cinato et al., 2024).

**TABLE 1:** Changes of HCM metabolites.

Metabolite	Trend of change	Main findings	References
Lactic acid	↑	It causes acidosis of myocardial cells and inhibits the calcium-regulating protein SERCA, thereby leading to calcium cycle disorders and systolic dysfunction	Newton et al. (2016), Deidda et al. (2021)
Acetyl coenzyme A	↓	The metabolites related to the tricarboxylic acid cycle decrease, as well as the enzymes decrease and their activities decline	Ranjbarvaziri et al. (2021), Previs et al. (2022)
ROS	↑↑	It causes oxidative damage to mitochondria and DNA	Wijnker et al. (2019), Lombardi et al. (2020)
BCAAs	↑	Continuous activation of mTOR promotes protein synthesis and translation, accelerating myocardial hypertrophy	Jørgenrud et al. (2015)
Ceramide and sphingomyelin	↑	Induce apoptosis of myocardial cells and the occurrence of atherosclerosis and myocardial infarction	Ranjbarvaziri et al. (2021), Jose et al. (2024)
Ketone bodies and their metabolites	↑	Increase myocardial energy intake	Deidda et al. (2021)

## 4 Glucose metabolism

In the pathological state of HCM, cardiomyocyte energy metabolism is marked by an increased reliance on glycolysis and a decreased capacity for aerobic glucose oxidation (Pereira et al., 2013; Zhao et al., 2025). This shift in metabolism reduces ATP production efficiency, contributing to the development of heart failure (Neubauer, 2007). At the molecular level, HCM cardiomyocytes exhibit altered expression of glucose transporter proteins. GLUT1 (SLC2A1), an insulin-independent transporter, is upregulated, while GLUT4 (SLC2A4), which is insulin-sensitive, is downregulated. This imbalance enhances glucose uptake despite the impaired oxidative metabolism. Concurrently, the activities of key glycolytic enzymes, such as hexokinase (HK) and phosphofructokinase (PFK), are significantly increased. However, the rate of glucose oxidation does not correspondingly rise, leading to an imbalance between glycolysis and oxidative metabolism (Shao and Tian, 2015). Myocardial glucose metabolism in HCM patients shows significant spatial heterogeneity. Glucose uptake is notably increased in areas with septal thickening and the lateral wall of the left ventricle, likely due to myocardial ischemia, microvascular disease, and local inflammatory responses, which disrupt normal blood supply and demand. Further studies have shown that myocardial glucose uptake correlates with biomarkers such as high-sensitivity troponin, left ventricular diastolic function parameters, and brain natriuretic peptide levels (Uehara et al., 1998; Aoyama et al., 2017). Additionally, the strong link between microangiopathy and myocardial fibrosis (Villa et al., 2016) is supported by cardiac magnetic resonance imaging, which reveals mild pathological glucose uptake in areas of myocardial hypertrophy (Barrio et al., 2021). These findings suggest that abnormal glucose metabolism could serve as a predictor of myocardial fibrosis risk in HCM patients.

In HCM, upregulation of the glycolytic pathway increases lactate production, resulting in intracellular acidosis in cardiomyocytes (Deidda et al., 2021). Acidosis impairs the function of sarcoplasmic reticulum calcium-ATPase (SERCA2a), a key enzyme responsible for calcium reuptake. As a result, calcium cycling is disrupted, leading to impaired cardiomyocyte contractility (Newton et al., 2016; De Michieli et al., 2024). The resulting abnormal calcium transients and delayed clearance contribute to diastolic dysfunction, a hallmark of early-stage HCM (Nakamura and Sadoshima, 2018). Over time, sustained diastolic dysfunction promotes pathological hypertrophy and structural remodeling of cardiomyocytes.

Glucose-6-phosphate, a key intermediate in glucose metabolism, regulates carbohydrate- and insulin-mediated cell growth by activating the mammalian target of rapamycin complex 1 (mTORC1) (Sharma et al., 2007; Sen et al., 2013). This highlights an intrinsic link between the glycolytic pathway and the proliferation and hypertrophy of cardiomyocytes. Adenosine monophosphate-activated protein kinase (AMPK), a key energy-sensing molecule, negatively regulates the mTOR pathway through phosphorylation. AMPK also promotes mitochondrial biosynthesis, boosts ATP generation efficiency, and suppresses energy-intensive biosynthetic pathways (Hardie et al., 2012). Given AMPK’s central role in energy metabolism, it has emerged as a promising therapeutic target for heart failure. This offers new avenues for treating cardiac diseases, including HCM (Kim and Dyck, 2015).

## 5 Lipid metabolism

Abnormal lipid metabolism plays a central role in the pathophysiology of HCM and is a well-established area of research (Doenst et al., 2013; Tan et al., 2021). Sara Ranjbarvaziri et al. demonstrated that the concentration of FFAs is elevated in HCM patients. Once inside cardiomyocytes, FFAs are converted to acyl coenzyme A and then to acyl carnitine (AC). AC enters mitochondria for β-oxidation via the carnitine shuttle system. Metabolic profiling of HCM myocardial tissue revealed a significant reduction in all types of ACs, particularly long-chain ACs (carbon chain length > C14), suggesting a metabolic block in the conversion of FFA to AC (Ranjbarvaziri et al., 2021). Gene expression analysis showed downregulation of key enzymes involved in fatty acid β-oxidation, including ultra-long-chain acyl-coenzyme A dehydrogenase, acyl-coenzyme synthetase long-chain family member 1, and hydroxyacyl-coenzyme A dehydrogenase (Ranjbarvaziri et al., 2021; Schuldt et al., 2021; Previs et al., 2022). Additionally, enzymes such as flavoprotein dehydrogenase and lipoacyl-coenzyme A hydratase one were also significantly downregulated. Patients with severe early-onset ultra-long-chain acyl-coenzyme A dehydrogenase deficiency often present with arrhythmias and myocardial hypertrophy, features commonly observed in HCM imaging (Strauss et al., 1995). Under stress from increased energy demand, these patients are prone to severe metabolic disorders, potentially leading to coma or death. Animal models have confirmed that ultra-long-chain acyl-coenzyme A dehydrogenase deficiency directly impairs cardiac function in mice (Saudubray et al., 1999). The fatty acid receptor CD36, which mediates FFA recognition and transport across membranes, exhibits significantly reduced activity in cardiomyocytes from HCM patients (Tucci et al., 2014).

Cardiomyocytes from patients with HCM exhibit disrupted sphingolipid metabolism, characterized by abnormal accumulation of ceramide and sphingomyelin (Jose et al., 2024). Ceramide, a central intermediate in sphingolipid metabolism, triggers apoptotic signaling in cardiomyocytes. Its accumulation correlates positively with disease progression and an increased risk of adverse cardiovascular events in HCM (Floegel et al., 2018). Mechanistic studies revealed significant downregulation of carnitine palmitoyltransferase I (CPT-I) in HCM cardiomyocytes (Lai et al., 2014). This impairs the transport of acyl-CoA into mitochondria for β-oxidation via the carnitine shuttle, causing a metabolic shift that channels excess fatty acids into sphingolipid biosynthesis pathways, leading to ceramide and sphingomyelin accumulation (Previs et al., 2022). The resulting sphingolipid accumulation contributes to cardiomyocyte apoptosis and may exacerbate structural remodeling in HCM. Additionally, it may promote atherosclerosis and elevate the risk of myocardial infarction.

Under normal conditions, ketone bodies contribute approximately 10% of the heart’s total energy supply and serve as auxiliary metabolic substrates. In cardiomyocytes, ketone bodies are metabolized into acetyl-CoA within mitochondria, which then enters the tricarboxylic acid (TCA) cycle to fuel oxidative phosphorylation (Cotter et al., 2013; Lopaschuk, 2017). Studies have reported significantly elevated levels of ketone bodies, including β-hydroxybutyrate, in cardiomyocytes from HCM patients. These elevations are inversely correlated with the expression of long-chain fatty acid oxidase, suggesting a metabolic shift away from fatty acid utilization. In a cohort study of patients with heart failure, elevated serum ketone body levels were associated with myocardial contractile dysfunction and increased circulating free fatty acids. These changes were accompanied by compensatory upregulation of key enzymes involved in ketone body metabolism (Aubert et al., 2016; Bedi et al., 2016). These findings suggest that increased ketone body utilization in HCM represents an adaptive metabolic response to impaired fatty acid oxidation and resultant energy deficiency.

## 6 Amino acid metabolism

In HCM, protein metabolism in cardiomyocytes and plasma exhibits significant abnormalities, with elevated levels of BCAAs, including leucine, isoleucine, and valine (Jørgenrud et al., 2015). BCAAs serve as crucial nitrogen and energy substrates. They activate the mTOR signaling pathway to support protein synthesis and participate in mitochondrial oxidative metabolism to meet cardiac energy demands. In the context of HCM, abnormal accumulation of BCAAs and their metabolites can lead to insulin resistance (Jang et al., 2016). Furthermore, the lysine metabolite aminocaproic acid is significantly elevated in HCM patients, and its levels correlate with cardiac remodeling processes (Jansen et al., 2023). In heart failure, the function of key enzymes in BCAA metabolism is impaired, primarily due to abnormal transcriptional reprogramming mediated by KLF15. This disruption results in elevated levels of branched-chain α-keto acids, which inhibit mitochondrial respiratory chain function and promote ROS production, including superoxide (Sun et al., 2016). Analyzing the dynamic changes in the amino acid metabolic network and its regulatory mechanisms in HCM will provide crucial insights into the disease’s pathogenesis and offer potential therapeutic targets for intervention.

## 7 Mitochondrial function and tricarboxylic acid cycle disorders

Mitochondria in HCM cardiomyocytes exhibit significant ultrastructural abnormalities, including swelling, disorganization, and reduced cristae density, compared to normal cardiomyocytes. These morphological abnormalities correlate with the downregulation of key genes involved in mitochondrial membrane organization, respiratory chain complex assembly, and cristae formation (Wijnker et al., 2019). These alterations impair mitochondrial oxidative phosphorylation and reduce respiratory chain complex activity, leading to decreased energy production in cardiomyocytes. Additionally, they disrupt intracellular oxidative-antioxidative homeostasis (Szyguła-Jurkiewicz et al., 2021). Mitochondrial oxidative stress is further aggravated by damage to the phospholipid-rich mitochondrial membrane (Liu and Chen, 2017). Under normal physiological conditions, ROS play essential roles in processes such as cardiac development, cardiomyocyte maturation, and calcium signaling, contributing to excitation-contraction coupling and vascular tone regulation (Peoples et al., 2019).

In HCM, elevated ROS levels cause oxidative damage to mitochondrial DNA, proteins, and lipids. This activates the mitochondrial permeability transition pore, induces mitochondrial dysfunction, and ultimately leads to cell death, disrupting the balance between intracellular ATP and ADP (Wijnker et al., 2019; Lombardi et al., 2020).

Dysfunction of the mitochondrial respiratory chain is a central pathological feature of HCM, contributing to impaired energy production and cellular dysfunction (Nollet et al., 2023). Studies have shown that in HCM cardiomyocytes, the efficiency of oxidative phosphorylation in respiratory chain complex V is significantly reduced. Additionally, the activity of complexes III and IV is also impaired, while the transcriptional levels of several mitochondrial complex components are downregulated (Nollet et al., 2023). Nollet et al. found that NAD + homeostasis is disrupted in myocardial samples from HCM patients, leading to impaired bioenergetic metabolism due to decreased efficiency in NADH conversion (Nollet et al., 2023). Despite compensatory increases in the precursor β-nicotinamide mononucleotide, the efficiency of the oxidative respiratory chain remains significantly impaired, suggesting that the compensatory mechanism is insufficient to restore normal mitochondrial function (Previs et al., 2022). Furthermore, decreased mitochondrial NAD + levels can lead to aberrant mitochondrial protein acetylation, disrupting normal mitochondrial function and contributing to cellular dysfunction in HCM (Lee et al., 2016). The conversion of nicotinamide to β-nicotinamide mononucleotide and β-nicotinamide mononucleotide to nicotinamide adenine mononucleotide is ATP-dependent. Therefore, abnormalities in the respiratory chain hinder the timely replenishment of ATP in cardiomyocytes, further exacerbating the energy depletion observed in HCM patients.

The tricarboxylic acid (TCA) cycle is a central metabolic pathway that facilitates the metabolism of carbohydrates, lipids, and proteins to supply energy in the body. Recent studies have shown a significant decrease in acetyl-CoA expression in the hearts of HCM patients compared to normal cardiac tissues (Previs et al., 2022). In HCM, the TCA cycle exhibits multiple metabolic abnormalities, including a reduced abundance of key circulating metabolites and decreased expression and activity of essential metabolic enzymes. In particular, the expression of pyruvate dehydrogenase is downregulated, and the catalytic activity of citrate synthase is significantly reduced. Additionally, the expression levels of malic enzyme 1, pyruvate carboxylase, and key intermediates of the TCA cycle, such as malic, citric, and succinic acids, as well as their encoding genes, are downregulated to varying extents (Ranjbarvaziri et al., 2021). These metabolic disturbances indicate significant defects in the metabolic pathways of carbohydrates, lipids, and proteins, as well as the TCA cycle, in the pathological process of HCM. These metabolic abnormalities severely impair myocardial energy production and contribute significantly to myocardial energy depletion in HCM patients.

## 8 Innovative research directions and therapeutic prospects

### 8.1 Application of metabolomics and molecular biology techniques

Metabolomics and advanced molecular biology techniques serve as robust tools for further elucidating the metabolic pathogenesis of HCM. By identifying metabolic signatures in bodily fluids and tissues, metabolomics provides diagnostic and pathological classification utility for HCM. At the plasma level, a large-scale metabolomics analysis of 441 HCM cases, 160 left ventricular hypertrophy (LVH) cases, and 119 healthy controls demonstrated significant differences in plasma metabolomes between HCM patients and both LVH patients and healthy individuals. Acylcarnitines, particularly C14:0-carnitine, displayed exceptional discriminatory power, achieving an area under the curve (AUC) of 0.937 on the receiver operating characteristic (ROC) curve for distinguishing HCM from LVH. This diagnostic accuracy was maintained at 0.83–1.0 in an independent validation cohort (Cui et al., 2025). Myocardial tissue metabolomics further identified HCM-specific pathological metabolic features: both genotype-positive (G+) and genotype-negative (G-) HCM patients exhibited shared characteristics, including inhibited fatty acid oxidation pathways and decreased lipid storage. These alterations were accompanied by compensatory elevations in alternative energy sources, such as 3-hydroxybutyrate and BCAAs. Concurrently, significant depletion of high-energy reserves, such as ATP and phosphocreatine (PCr), directly correlated with diastolic dysfunction indicators, including left atrial enlargement (Nollet et al., 2024). Furthermore, multi-omics integration confirmed paradoxical features in HCM myocardium, including free fatty acid accumulation alongside reduced long-chain acylcarnitines, as well as oxidative stress signals indicated by an elevated oxidized glutathione/reduced glutathione ratio. These elements collectively constitute a metabolic fingerprint distinct from other cardiac diseases. Notably, this signature is detectable even in early-stage HCM patients with preserved left ventricular ejection fraction, underscoring the early diagnostic specificity of metabolomics (Ranjbarvaziri et al., 2021; Previs et al., 2022).

### 8.2 Potential therapeutic targets and intervention strategies

Key metabolic nodes in HCM offer opportunities for developing novel therapeutic strategies. In fatty acid metabolism pathways, HCM myocardium typically exhibits impaired β-oxidation, with long-chain acylcarnitine levels reduced by 70%–95%. This impairment may be ameliorated by modulating carnitine palmitoyltransferase I (CPT-I) activity to restore fatty acid transport and mitochondrial oxidation, while avoiding agents such as trimetazidine that further inhibit fatty acid oxidation and exacerbate energy deficits (Ranjbarvaziri et al., 2021). Regarding the paradox of FFAs accumulation coupled with downregulated transporter expression (CD36, SLC27A4), interventions targeting transporter membrane localization warrant exploration, rather than solely increasing total protein levels. For alternative energy utilization, the compensatory reliance of HCM myocardium on 3-hydroxybutyrate and BCAAs suggests that energy supply could be enhanced through supplementation with ketone precursors or activation of the branched-chain α-ketoacid dehydrogenase complex. Such approaches may reduce BCAAs accumulation and their toxic metabolites, thereby mitigating insulin resistance and myocardial hypertrophy. Restoring mitochondrial function represents a critical therapeutic target. To counteract reduced activity in respiratory chain complexes (II, V) and disrupted NAD^+^ homeostasis, supplementation with β-nicotinamide mononucleotide (NMN) can replenish NAD^+^ levels, whereas coenzyme Q10 can enhance respiratory chain efficiency. Concurrently, activating mitochondrial autophagy—via upregulation of BNIP3 and PINK1 expression—can eliminate damaged mitochondria exhibiting disrupted cristae structures, thereby attenuating oxidative stress (Previs et al., 2022). Exacerbated oxidative stress substantially contributes to metabolic dysregulation in HCM. Supplementation with reduced glutathione (GSH) or mitochondrial-targeted antioxidants can decrease myocardial phospholipid oxidation, thus preserving mitochondrial membrane integrity. Notably, combined metabolic interventions may achieve superior therapeutic outcomes compared to single-target approaches. Future clinical trials are essential to evaluate the safety and efficacy of these combined strategies.

## 9 Conclusion

**FIGURE 1:**
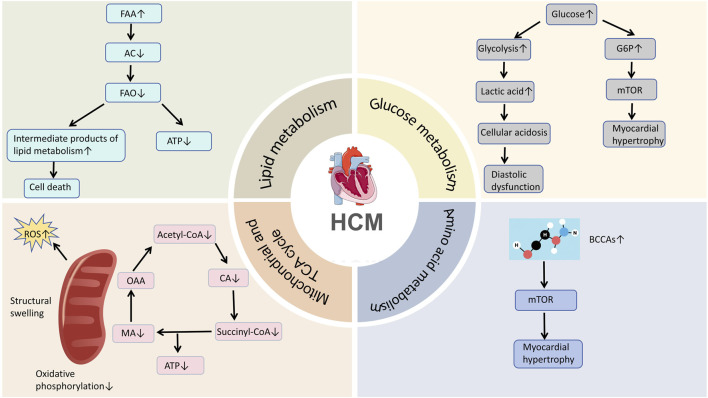
Summary of this article.

Cardiac metabolic reprogramming in HCM patients is a multifaceted pathological process. It involves key metabolic domains, including glucose metabolism, lipid metabolism, amino acid metabolism, and mitochondrial function regulation (Figure 1). This metabolic reprogramming contributes significantly to cardiac failure by disrupting the cardiac energy supply system, impairing efficient energy production in cardiomyocytes. In HCM, metabolic reprogramming is not just a byproduct of disease progression but may also serve as a critical marker of cardiac adaptive dysregulation. Systemic alterations in metabolic pathways disrupt energy homeostasis in cardiomyocytes, leading to both systolic and diastolic dysfunction in the heart. Moreover, as a central organ in metabolic regulation, the heart’s abnormal metabolic shifts can significantly impact systemic metabolic homeostasis through alterations in metabolic signaling. Understanding the molecular mechanisms and regulatory network of cardiac metabolic transitionin HCM not only clarifies the pathophysiology of the disease but also lays the groundwork for developing novel therapeutic strategies and intervention targets. In the future, targeting key cardiac metabolic pathways could significantly improve the clinical outcomes and quality of life for HCM patients.
