# Correction: Mechanisms of metabolic transition in hypertrophic cardiomyopathy

**DOI:** 10.3389/fphys.2025.1764781

**Published:** 2026-01-09

**Authors:** Conghao Tan, Zhexuan Guo, Junjie Zhou, Wei Yuan

**Affiliations:** 1 Department of Cardiology, Affiliated Hospital of Jiangsu University, Zhenjiang, China; 2 Institute of Cardiovascular Diseases, Jiangsu University, Zhenjiang, China

**Keywords:** hypertrophic cardiomyopathy, metabolic transition, mitochondrial dysfunction, glucose metabolism, lipid metabolism

**Affiliation** Institute of Cardiovascular Diseases, Jiangsu University, Zhenjiang, China was omitted for authors Conghao Tan, Zhexuan Guo, Junjie Zhou and Wei Yuan. This affiliation has now been added for authors Conghao Tan, Zhexuan Guo, Junjie Zhou, Wei Yuan.

**Affiliation** Department of Cardiology, Affiliated Hospital of Jiangsu University, Zhenjiang, China was erroneously given as Department of Cardiovascular Medicine, Zhenjiang First People’s Hospital, Zhenjiang, Jiangsu, China.

The original article has been updated.

